# Mapping Wintering Waterfowl Distributions Using Weather Surveillance Radar

**DOI:** 10.1371/journal.pone.0041571

**Published:** 2012-07-23

**Authors:** Jeffrey J. Buler, Lori A. Randall, Joseph P. Fleskes, Wylie C. Barrow, Tianna Bogart, Daria Kluver

**Affiliations:** 1 Department of Entomology and Wildlife Ecology, University of Delaware, Newark, Delaware, United States of America; 2 National Wetlands Research Center, United States Geological Survey, Lafayette, Louisiana, United States of America; 3 Western Ecological Research Center, United States Geological Survey, Dixon, California, United States of America; 4 Department of Geography, University of Delaware, Newark, Delaware, United States of America; University of Osnabrueck, Germany

## Abstract

The current network of weather surveillance radars within the United States readily detects flying birds and has proven to be a useful remote-sensing tool for ornithological study. Radar reflectivity measures serve as an index to bird density and have been used to quantitatively map landbird distributions during migratory stopover by sampling birds aloft at the onset of nocturnal migratory flights. Our objective was to further develop and validate a similar approach for mapping wintering waterfowl distributions using weather surveillance radar observations at the onset of evening flights. We evaluated data from the Sacramento, CA radar (KDAX) during winters 1998–1999 and 1999–2000. We determined an optimal sampling time by evaluating the accuracy and precision of radar observations at different times during the onset of evening flight relative to observed diurnal distributions of radio-marked birds on the ground. The mean time of evening flight initiation occurred 23 min after sunset with the strongest correlations between reflectivity and waterfowl density on the ground occurring almost immediately after flight initiation. Radar measures became more spatially homogeneous as evening flight progressed because birds dispersed from their departure locations. Radars effectively detected birds to a mean maximum range of 83 km during the first 20 min of evening flight. Using a sun elevation angle of −5° (28 min after sunset) as our optimal sampling time, we validated our approach using KDAX data and additional data from the Beale Air Force Base, CA (KBBX) radar during winter 1998–1999. Bias-adjusted radar reflectivity of waterfowl aloft was positively related to the observed diurnal density of radio-marked waterfowl locations on the ground. Thus, weather radars provide accurate measures of relative wintering waterfowl density that can be used to comprehensively map their distributions over large spatial extents.

## Introduction

The current United States network of weather surveillance radars known as WSR-88D (Weather Surveillance Radar 1988 Doppler) or NEXRAD (NEXt generation RADar) readily detects biological targets aloft (e.g., birds, bats, and arthropods), and has proven to be a useful remote-sensing tool for ornithological study [1]–[5]. Weather radars provide broad spatial and temporal scale information that can help answer questions about how bird movement patterns, habitat use, or numbers are affected by physiographic features (e.g., large bodies of water, mountain ranges, deserts), land cover changes, habitat management, weather, climate change, and other factors [6].

One application of weather radar has been to map the distributions of landbirds, at the ground during migratory stopover by measuring the strength of radar echoes (i.e., reflectivity) produced by birds upon leaving stopover areas at the onset of nocturnal migratory flight [5], [7]–[10]. Because nocturnal migratory flights are closely synchronized to the elevation of the sun [11]–[13], birds are typically sampled using a single near-instantaneous radar scan collected during the abrupt *en masse* exodus of birds on a given night. Buler and Diehl [10] used data collected at evening exodus to demonstrate that radar reflectivity measures are strongly correlated with ground observations of migrant landbird densities, and provide relative bird density measures that can be quantitatively compared across the radar area after being adjusted for biases caused by the height distribution of birds in the air and radar beam geometry.

Like migratory landbirds, wintering waterfowl engage in routine, synchronized feeding flights. Field-feeding species such as mallards (*Anas platyrhynchos*) and northern pintails (*A. acuta*) regularly engage in flights between habitats used mainly for resting and those used for feeding [14]. Dynamics of these feeding flights have been studied throughout North America, including the agricultural/wetland habitat systems of the West Gulf Coastal Plain and the Central Valley of California (CVC). Although there is interspecific, geographic, and intraseasonal variability in the exact timing of these feeding flights (e.g., [15], [16]), these movements tend to occur twice a day at dawn and dusk during the wintering period and are closely synchronized to sun elevation [17]–[20]. For example, Cox and Afton [20] noted that the evening flights of northern pintails in Louisiana averaged 22 minutes after sunset, while Baldasarre and Bolen [18] observed that the evening departures of several field-feeding duck species wintering in Texas averaged 25±2 (SE) min after sunset.

Given that evening feeding flights are well synchronized and radar reflectivity is positively correlated to the density of waterfowl aloft [21], [22], weather radar could also be used to map the diurnal distributions of wintering waterfowl. However, the development and evaluation of such an approach has not been done. Developing an approach to observe wintering waterfowl distributions at broad landscape and regional scales using weather radar data has important implications for conservation and management planning. For example, the analysis of radar data could help build a better understanding of the juxtaposition of diurnal roosts and feeding habitats critical to effectively conserve and manage landscapes for wintering waterfowl [23], [24].

A careful, empirical examination of how the distribution of birds in the airspace changes over time during the onset of synchronized flight as observed by weather radar has not been done. Such an examination would be useful for determining an optimal instantaneous sampling time of radar observations of birds aloft to map their estimated distributions at the ground. The optimal sampling time should balance conflicts between data accuracy and the effective sampling area of the radar. For example, as waterfowl fly farther away from their departure locations, the locational accuracy of radar measures of their distributions aloft will worsen relative to their original distributions on the ground. Furthermore, waterfowl increasingly disperse into the air during flight. The appearance of the resulting sequence of radar scans due to this dispersion is akin to turning the focus knob when looking through binoculars and blurring the view; radar measures become more homogenous and the extent of spatial autocorrelation in measures increases. This resulting loss of spatial detail when mapping bird distributions makes it more difficult to quantify differences in bird densities among ground sources [8]. Thus, sampling birds aloft as close to the initiation of flight as possible maximizes the accuracy and spatial heterogeneity and minimizes the extent of spatial autocorrelation of the data. The trade-off to this approach, however, is that it also minimizes the range out to which the radar can detect birds since there is a positive correlation between the height of birds above the ground and the distance the radar is able to detect them. This is because the height of the radar beam above the ground increases as it propagates out from the radar antenna. If the height of birds in the air increases rapidly at the onset of evening flights, the detection range can be increased by sampling waterfowl later during the evening flight period, allowing the mapping of bird distributions over a greater area. Thus, an optimal sampling time can be assessed by measuring 1) the correlation between radar-measured bird density aloft and observed bird density on the ground, 2) the spatial heterogeneity and extent of spatial autocorrelation of radar measures, and 3) the maximum effective distance that the radar can detect birds.

Assessing the accuracy of radar measures requires data of known bird densities on the ground over a large spatial extent. Previously, Fleskes et al. [25] tracked day and night locations of individual radio-marked northern pintail and mallard within the northern CVC. These two species comprised 52% (36% and 16%, respectively) of all dabbling ducks observed within the northern CVC during the studied winters and were concentrated in the same general locations as most other waterfowl species based on mid-winter surveys conducted by California Department of Fish and Game and U.S. Fish and Wildlife Service. Thus, diurnal distributions of mallards and pintails would likely be representative of the diurnal distribution of most other waterfowl and locations of the radio-marked ducks span the radar areas of two WSR-88D stations. This telemetry dataset provides a unique opportunity for assessing weather radar observations for mapping waterfowl distributions.

Our objective was to adapt, improve, and validate the approach of Buler and Diehl [10], for mapping the diurnal distributions of wintering waterfowl using weather surveillance radar. We also present an empirical analysis of the determination of an optimal instantaneous sampling time for maximizing the usefulness of radar-based bird distribution maps in general. For this, we evaluated the accuracy and precision of radar-based bird density maps created at different time points during the course of evening flight relative to observed diurnal distributions of radio-marked waterfowl on the ground. We also evaluated the change over time in the height distribution of waterfowl in the air and the effective maximum range at which the radar detected birds. We validated our approach by assessing the association between radar data from two weather surveillance radars and the diurnal density of radio-marked mallards and pintails on the ground among two winters.

### Study Area

**Figure 1:**
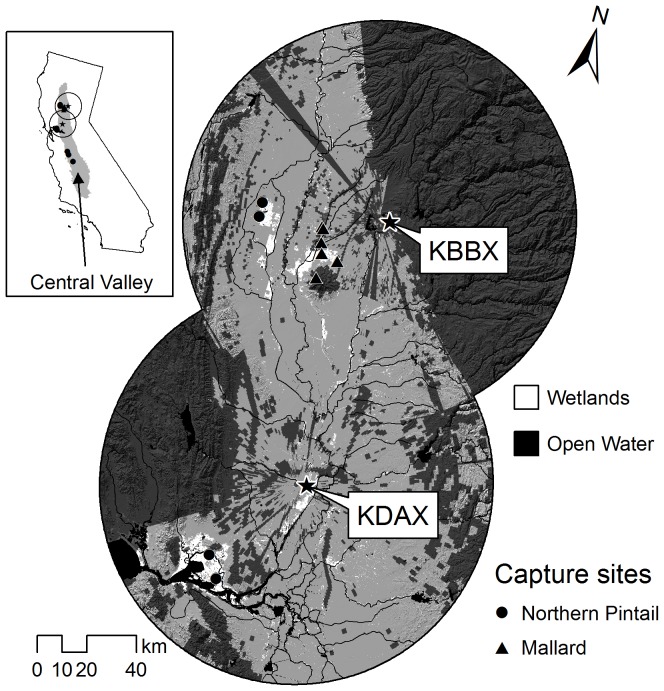
Study area within the Central Valley of California. Locations (stars) and names of weather radars and their associated 80 km radius surveillance areas are shown. Also shown are capture locations of radio-marked northern pintail and mallard during winters of 1998–1999 and 1999–2000. Dark grey-shaded areas denote where radar data were masked because of persistent ground clutter contamination or partial radar beam blockage.

We used data from two WSR-88D stations (KDAX; 38.50111°N, 121.67778°W, and KBBX; 39.49611°N, 121.63167°W) located near Sacramento, California that provide radar coverage of the northern half of the CVC (Figure 1). The CVC provides critical wintering habitat for many species of waterfowl in the Pacific Flyway. Agricultural and human development have reduced the extent of the estimated 1.6 to 2 million hectares of original wetlands in the CVC by over 90%. Many wetlands in the northern CVC were converted to rice, corn, or other grain that have high forage value to waterfowl [26], resulting in a landscape where waterfowl roost on wetlands and feed in surrounding croplands.

## Methods

### Weather surveillance radar data

We obtained radar data collected during the period of peak wintering waterfowl population numbers (December 1 through January 31; [25]) for the winters of 1998–99 and 1999–2000 for KDAX , and winter of 1998–99 for KBBX from the data archive of the National Oceanic and Atmospheric Administration's (NOAA) National Climatic Data Center (NCDC; http://www.ncdc.noaa.gov/nexradinv/). Radar data from winter 1999–2000 for KBBX were missing from the archive. We used Level II radar data that consist of measures taken within sampled volumes of air with dimensions of 1 km in length by 0.95° in diameter. Sample volumes are identified by their range from the radar and azimuth relative to true north. We used radar reflectivity factor, a measure of returned radar echo strength based on the dielectric properties of water in units of Z (mm^6^ m^−3^) and determined by the density and size of targets within the sampled volume, as a relative measure of bird density [1], [3], [27], [28]. We refer readers to Crum and Alberty [29] for details about the operational specifics of WSR-88D.

We visually screened radar data to exclude nights from consideration when any precipitation was present within 100 km of the radar or there was extreme refraction of the radar beam toward the ground (a.k.a. anomalous propagation) due to non-standard atmospheric conditions. Anomalous propagation results in contamination of reflectivity measures due to echo returns from the beam hitting the ground. Overall, we sampled evening waterfowl feeding flights from 30% (55 of 186) of potential days across winters and radar sites. We excluded the remaining 70% percent of days from analyses due to the presence of precipitation (28%), missing data in the archive (27%), or anomalous propagation (15%).

We locally interpolated reflectivity measures to the same relative time point with respect to sun elevation to reduce potential temporal sampling error and bias [10]. Temporal sampling error is due to the relatively-coarse sampling rate of WSR-88D (e.g., one radar scan every 6 or 10 min) and the lack of synchronization of sampling to the onset of bird flights. The sampling error among nights could be up to five minutes difference, which may be substantial given that the number of birds in the air doubles every ∼2.5 min for landbirds during the onset of nocturnal migratory flight [12], [13]. With regards to the sampling bias within scans, there is an approximately eight-minute time differential in the position of the sun across the radar domain along an east-west axis. Thus, waterfowl at the eastern extent of the radar domain are likely sampled later in their feeding flight than waterfowl at the western extent. We interpolated reflectivity data by weighting the two measures collected immediately before and after the target time point when the sun elevation was 5.0° below horizon by the inverse of the time that they deviated from the target sampling time. This was the optimal target sun elevation for quantifying the relative diurnal density of waterfowl on the ground based on our analysis of the association of reflectivity measures with ground telemetry data presented later.

The radar beam spreads as it travels away from the radar, causing it to sample the air at different heights at different ranges from the radar. This differential sampling of the distribution of birds in the air precludes the direct comparison of original radar measures at different ranges and at different ground elevations. We accounted for this sampling bias by adjusting interpolated radar data using the algorithm of Buler and Diehl [10]. The algorithm adjusts reflectivity measures of individual sample volumes to a common reference; the estimated mean reflectivity of birds in the air from the ground up to 1750 m above ground level (AGL). This allows for the direct comparison of measures throughout the radar coverage area. We improved the algorithm by using a Gaussian (rather than uniform) power distribution of the beam [30] and by extending the width of the beam from the typically-considered 0.95° (3-dB) to 1.37° (6-dB), which incorporates 93.75% of the total power of the radar beam [31]. As part of the data processing algorithm, reflectivity measures are resampled into a fixed reference grid of quarter-degree-wide sample volumes (1 440 azimuths at each range). This is because, as an analog instrument, there is slight variability in the azimuth sampling scheme among different radar scans. All further mention of “sample volumes” refers to these fixed quarter-degree sample volumes.

For each radar site, we produced a masking map of the individual sample volumes where reflectivity measures were regularly influenced by persistent ground clutter contamination or partial radar beam blockage from human infrastructure or topography. Data from masked sample volumes were excluded from all analyses. Regions of ground clutter and beam blockage are easily detected by determining the probability of detection (POD) over a long time period [32]. Accordingly, we produced POD maps using ∼4 000 volume scans collected during the month of June 1998 and 1999 when biological activity in the air was at an annual minimum. We identified ground clutter based on extremes in POD. Low POD is indicative of chronic clutter suppression applied by the WSR-88D operating system [33], while high POD is indicative of persistent unsuppressed ground clutter from moving ground targets (e.g., cars on highway overpasses, wind farms). We identified beam blockage from human infrastructure by a distinct and stable drop in POD along the entire azimuth beyond the ground source of the blockage. We also estimated the amount of beam blockage due to topography using the simplified beam interception function outlined by Bech et al. [34]. We determined mean ground height beneath sample volumes using the National Elevation Data set assembled by the U.S. Geological Survey. We excluded data from sample volumes with >30% of the radar beam blocked due to topography. Overall, approximately 20% of sample volumes were excluded from analysis due to regular clutter contamination or severe beam blockage.

As an additional quality control measure, we excluded data from individual sample volumes during each sampling night where the radar beam sampled ≤5% of the vertical distribution of birds in the air (i.e., sampled primarily empty airspace above birds) as determined by the algorithm of Buler and Diehl [10] to prevent inclusion of false zeros. We further excluded consideration of pulse volumes where data were excluded for ≥25% of sampling nights within a winter.

The culmination of our methodology development was the creation of the software package BIRDS (Bias Improvement of Radar Data System). This software is a system of Java scripts, Python scripts, and Fortran95 code operating in a UNIX environment and optimized for relatively fast processing time. After providing uncompressed raw radar data from the NCDC archive, BIRDS converts batched data (i.e., samples from multiple days) from one radar station to ASCII data, performs the interpolation (temporally and spatially) to a specified sun angle, adjusts reflectivity measures according to Buler and Diehl [10], and provides summary statistics for every sample volume. We developed the BIRDS software to facilitate radar data analyses. BIRDS is freely available for distribution upon request to the corresponding author.

### Radio telemetry data

We used 8,076 daytime (based on local sunset/sunrise) locations of waterfowl collected within 100 km of either radar site during December and January 1998–2000 (see [35] for details). Waterfowl were tracked daily from trucks or fixed-wing aircraft throughout the study period. Daytime locations were recorded once a day with an estimated precision of 1.1 ha [36]. Weekly aerial searches of waterfowl habitat and urban areas throughout the Central Valley were also conducted for missing radio-marked waterfowl. Locations were from a total of 365 individual northern pintail and mallard. The Animal Care and Use Committee of the USGS Western Ecological Research Center reviewed and approved our methods to ensure that they were in compliance with the Animal Welfare Act and United States Government Principles for the Utilization and Care of Vertebrate Animals Used in Testing, Research, and Training policies.

Adult (i.e., after first calendar year) female pintails (*n* = 261) were captured and radio-marked during late August through early October of each study year at three wetland locations; the Colusa Basin in the northern CVC, the Suisun Marsh just to the west of the CVC, and the Grassland Ecological Area and Mendota Wildlife Area in the southern CVC. The Colusa Basin and Suisun Marsh are within the combined radar coverage area (Figure 1), but individual pintails are highly mobile and were located, on average, 120.9±5.5 km from their capture site during the course of a winter.

Adult and juvenile (i.e., first calendar year) female mallards (*n* = 104) were captured and radio-marked during late August through mid-September of each study year at Graylodge and Upper Butte Basin Wildlife Area in the northern CVC near the KBBX radar. With an average location of 16.0±1.4 km from their capture site, individual mallards exhibited limited movement compared to pintails and their locations were almost exclusively restricted to within the KBBX radar area. Therefore, we excluded mallard locations for comparisons with radar data from KDAX.

The tracking schedule of radio-marked ducks was designed to adequately sample variation in distribution and movement patterns related to season, time of day, and variation in hunting activity while minimizing dependence among locations. Thus, locations were determined for each radio-marked duck several times each week during the day and night but relocation frequency for each was normally limited to one diurnal and one nocturnal location within each 24-hour period.

### Visual field observations

We visually monitored and made video recordings of bird flight activity from the ground at four different wetland locations throughout the study area among four evenings in January 2009. While these data were not collected concurrent with the radar data we analyzed, they provided information about the timing, behavior, and species of birds in flight before and during the onset of evening waterfowl feeding flights.

### Data analysis

We evaluated three factors during the onset of evening flights to determine which sun elevation angle to use when quantifying the relative diurnal density of waterfowl at the ground; 1) the spatial heterogeneity and autocorrelation of reflectivity measures, 2) the magnitude of the correlation between reflectivity and observed waterfowl density on the ground, and 3) the maximum effective detection range of birds aloft. We first assessed correlations between mean reflectivity and a range of kernel density estimates of radio-marked waterfowl locations produced using different bandwidth sizes (i.e., smoothing parameter values) for a series of time points during the onset of evening flight. We identified the bandwidth size that produced the strongest correlation between mean kernel waterfowl density and mean reflectivity for a given time point as the scale at which smoothing of waterfowl density on the ground produced the closest match to bird density aloft. This provided us a measure of how finely “focused” the radar data are relative to ground data throughout the onset of evening flight. We used the quadratic kernel function of Silverman [37] to create a 30-m-resolution raster grid surface of the estimated density among all radio-marked waterfowl locations during a winter, and then averaged kernel density values of grid cells located within the area boundaries of a sample volume to derive mean kernel density for each sample volume. We computed kernel density for bandwidth sizes ranging from 500 m to 10 km by 500 m intervals. To reduce the influence of spatial autocorrelation in the data, we drew random samples of 30 sample volumes that were separated by at least 10 km to generate 2 000 bootstrap samples. We then averaged correlation coefficients across the collection of bootstrap samples by radar, bandwidth, and winter. We log-transformed data to meet the assumption of a bivariate normal distribution.

Because the spatial heterogeneity of reflectivity measures is largely influenced by the displacement of birds from their departure location [8], we also estimated the median travel distance of birds aloft for each volume scan. We did this by multiplying the median height of birds aloft, based on their vertical distribution estimated from the radar data processing algorithm, by the product of the mean horizontal ground speed (17.5 m/s) and the mean vertical ascent rate (0.5 m/s) of waterfowl species during climbing flight [38], [39]. This calculation assumes birds fly linearly at a constant vertical and horizontal speed during these evening flights. This simplification of bird flight behavior ignores variability in the flight speeds of waterfowl due to wind [40] and potentially non-linear flight paths. Thus, there is an unknown and likely moderate degree of uncertainty in displacement distance estimates. However, these estimates are still informative in understanding the amount of spatial heterogeneity of the radar data.

We determined the scale of spatial autocorrelation of the radar data using semivariogram analysis [41]. We fit the empirical semi-variance among a random sample of 5000 adjusted reflectivity measures as a function of the distance between the centroids of sample volumes for each night using the following isotropic exponential function:
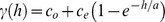
where *h* is the distance between two measures (i.e., the lag distance), *c_o_* is the variance due to sampling error and/or spatial dependence at distances smaller than the sampling interval, *c_e_* is the partial population variance, and *a* is the range. The modeled function quantifies how variance between radar measures changes as a function of the distance between them. The variance among measures increases as the lag distance between measures increases (i.e., spatial autocorrelation decreases). The range represents the distance at which spatial autocorrelation in the radar measures approaches its minimum. The assumptions of normality and stationarity of variance were met by log-transforming and detrending reflectivity measures. We detrended reflectivity values by fitting first-order polynomial models within a local moving window of 15 km radius.

We determined the mean maximum range that the radar detected birds for those azimuths where the radar beam was unobstructed by topography out to 100 km range (*n* = 308). The maximum detection range along each azimuth radial was the farthest distance at which a radar sample volume sampled at least 5% of the birds in the air for at least 75% of sampled nights within a winter.

## Results

**Figure 2:**
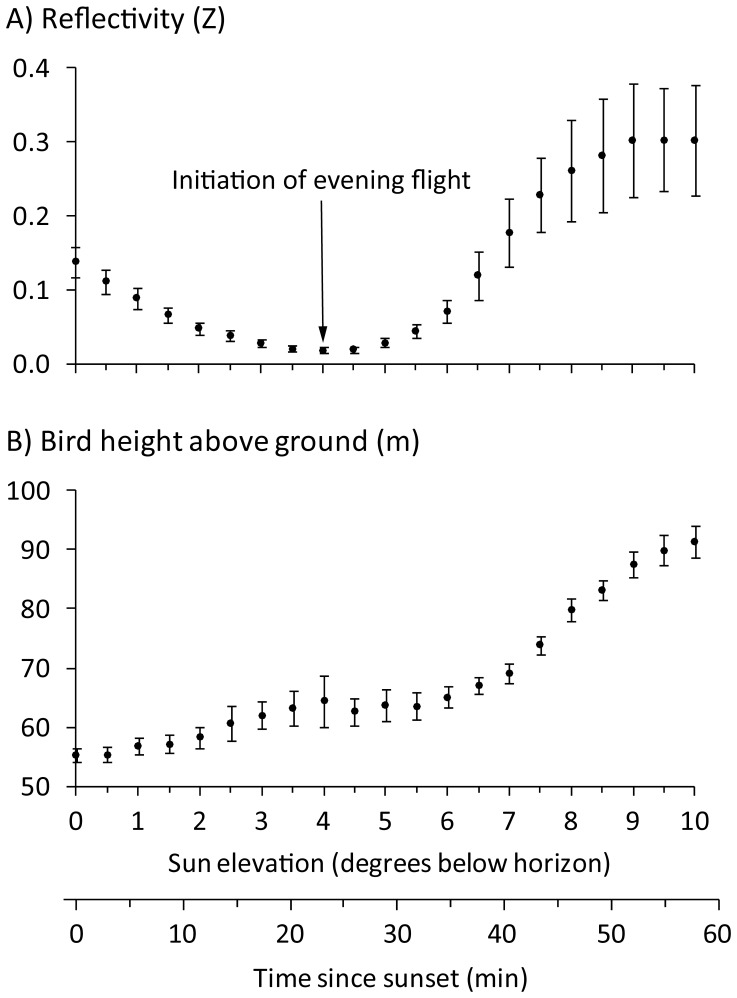
Radar measures of bird density and flight heights during evening flight. Time series depicting the change in A) mean radar reflectivity (i.e., index of bird density) and B) mean height of birds aloft during evening flights around the KDAX radar during the winters of 1998–1999 and 1999–2000 (*n* = 44 days). Error bars denote ±1standard error of the mean.

We analyzed the onset of evening flights for 44 days from the KDAX radar during winter 1998–1999 and 1999–2000. Beginning at sunset, the magnitude of mean reflectivity across days was relatively moderate (7 times greater than the minimum) and exhibited a stable shallow rate of decline over time (8% per min) until reaching its minimum value when sun elevation reached −4° (23 min after sunset) (Figure 2A). Based on our field observations from 2009, most of the birds flying at this time were greater white-fronted goose (*Anser albifrons*) and white-faced ibis (*Plegadis chihi*). The abundance and flight activity of these birds declined as they landed to roost within wetland habitats. From 23 min after sunset until the end of our sampling time window (56 min after sunset) mean reflectivity increased, indicating increasing relative bird density in the air. We considered the start of this increase in reflectivity as the mean initiation time for evening flights. Most of the birds we observed in 2009 participating in evening flights were waterfowl, comprised mostly of dabbling ducks like northern pintail and mallard. However, we also observed black-crowned night-herons (*Nycticorax nycticorax*) engaging in the evening flights. Flight initiation times varied among individual nights, ranging from 11 to 34 min after sunset with a standard deviation about the mean of 4 min. As evening flight progressed, the relative rate of change in mean reflectivity increased steeply until reaching a maximum of 24% per min at a sun elevation of −7° (40 min after sunset). Then the rate of change declined until mean reflectivity reached an asymptotic value 16 times greater than that at the initiation of feeding flight. The mean height of birds above ground derived using estimated vertical reflectivity profiles closely matched the change in reflectivity after the initiation of feeding flight, increasing from 64±4 m to 91±3 m (Figure 2B).

**Figure 3:**
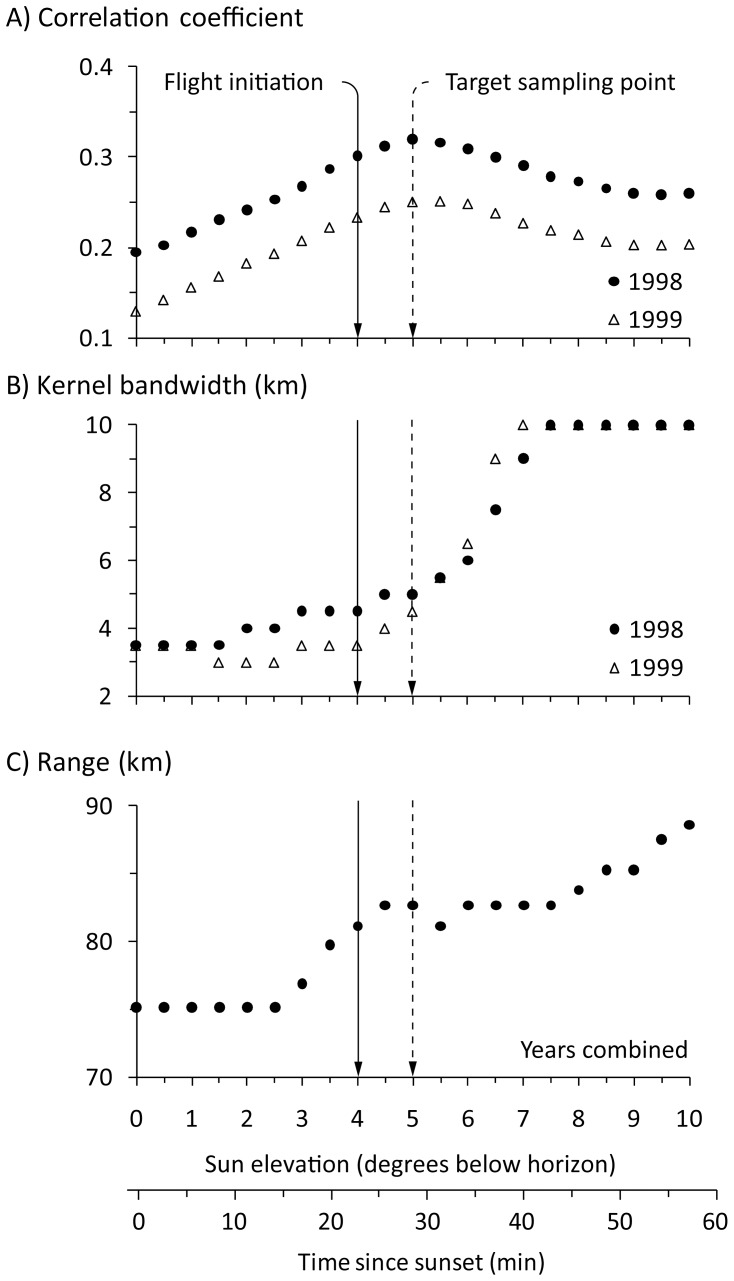
Accuracy, precision, and detection range of radar observations of birds during evening flight. Time series depicting the change in radar data accuracy, precision, and detection range as measured, respectively, by A) the mean correlation between mean radar reflectivity (i.e., index of bird density) and the mean kernel density of radio-marked waterfowl locations within a 0.5 km bandwidth, B) the kernel density bandwidth size of the maximum mean correlation coefficient between mean radar reflectivity and mean kernel density of radio-marked waterfowl locations, and C) the mean maximum range that the radar detected birds in the air around the KDAX radar among 2 000 bootstrapped samples of 30 individual sample volumes during the winters of 1998–1999 (*n* = 18 days) and 1999–2000 (*n* = 26 days). Standard errors for plots A and C were too small for display.

The sun elevation of −5.0° (i.e., 5 min after our estimated mean flight initiation time) was the optimal target sun elevation for quantifying the relative diurnal density of waterfowl at the ground. The mean reflectivity during each winter was most-strongly and positively correlated to the density of radio-marked waterfowl locations on the ground at a sun elevation of −5.0° (Figure 3A). At the initiation of evening flight, the kernel bandwidth distance of ground data that produced the strongest correlation with reflectivity was relatively fine among winters, but increased sharply in coarseness as flight progressed; reaching the maximum evaluated bandwidth of 10 km at a sun elevation of −7.5° (∼20 min after flight initiation) (Figure 3B). The overall mean maximum detection range of birds was stable and relatively moderate (∼83 km) for about 25 min after the initiation of evening flight before increasing thereafter (Figure 3C). During the time period where the maximum detection range remained relatively constant, median bird height averaged 110±1 m.

**Figure 4:**
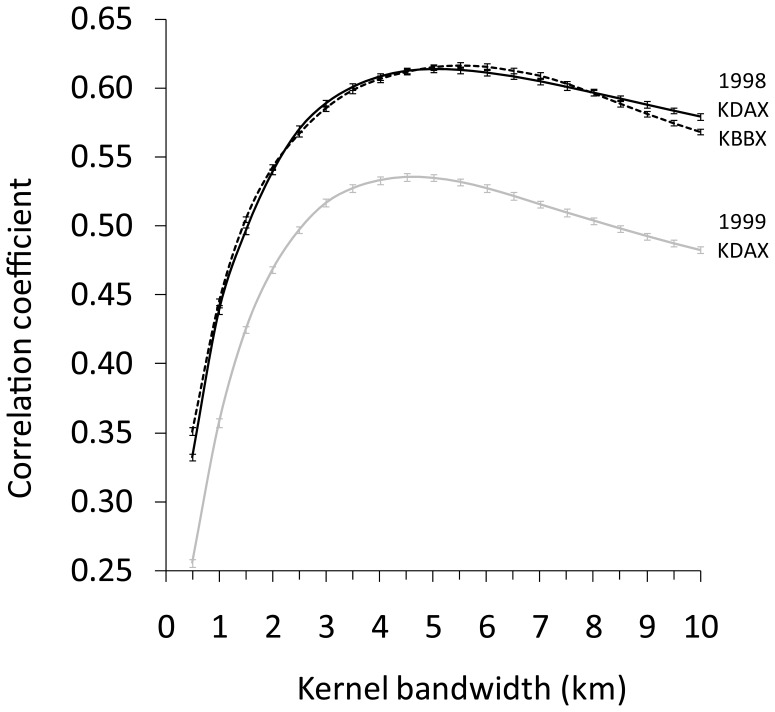
Correlations between radar and telemetry data. Mean correlation among 2 000 bootstrapped samples between the mean radar reflectivity (i.e., index of bird density) and the kernel density of radio-marked waterfowl locations estimated for different bandwidth sizes by winter and radar. Error bars denote ±1 standard error.

**Figure 5:**
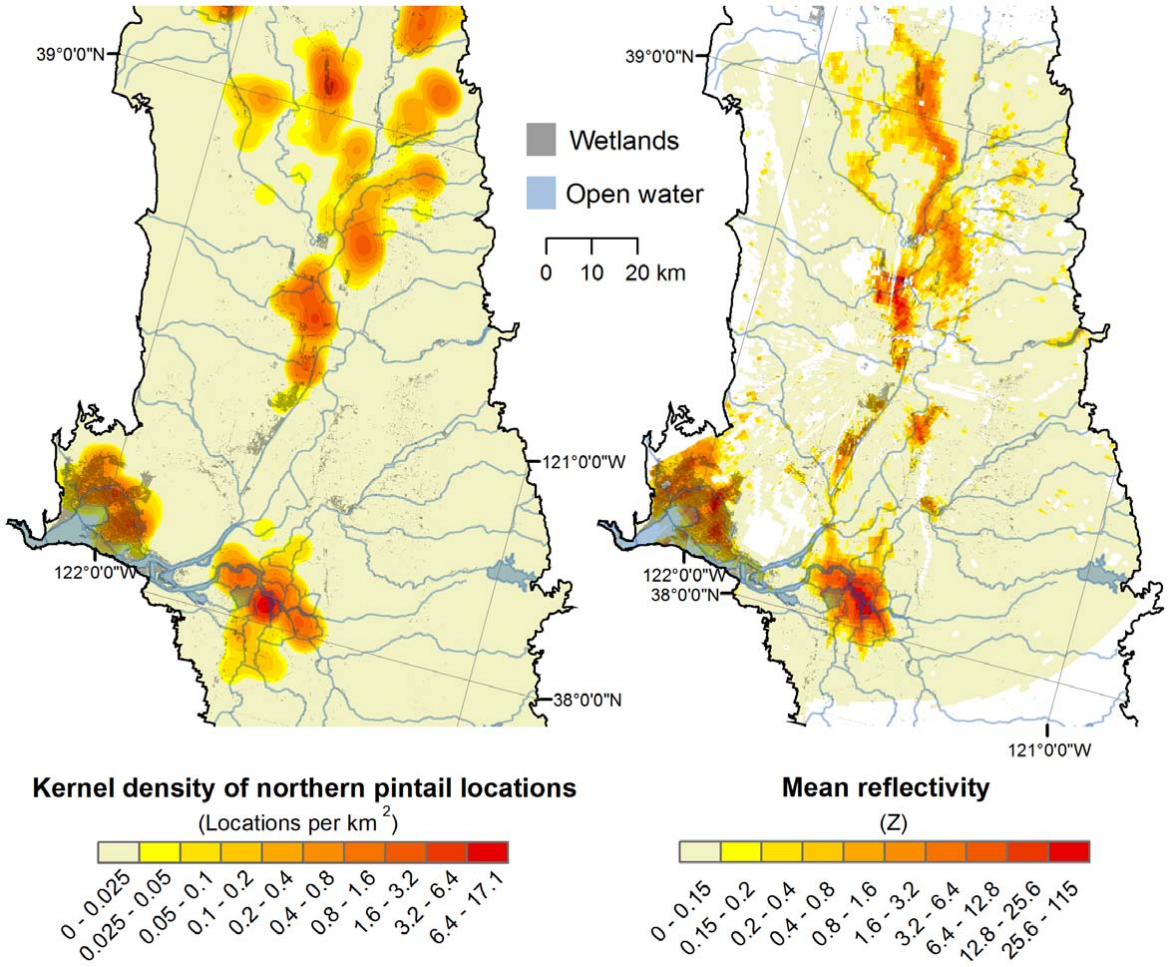
Maps of wintering waterfowl distributions. Maps of the 5-km-bandwidth kernel density of radio-marked northern pintail locations (*n* = 3 102) and the mean radar reflectivity (i.e., index of bird density) around the KDAX radar (*n* = 18 days) during winter 1998–1999. White areas denote regions where radar data were masked from analysis.

At a sun elevation of −5.0°, the strongest correlations between mean reflectivity and the kernel density of radio-marked northern pintails around the KDAX radar were 0.61 during winter 1998–1999 and 0.54 during winter 1999–2000 (Figure 4). The strongest correlation between mean reflectivity and the kernel density of radio-marked mallards and northern pintails around the KBBX radar during winter 1998–1999 was 0.62. There were close spatial associations between areas of the greatest reflectivities and radio-marked bird densities (Figure 5). We note that the radars also measured a few “hotspots” of high reflectivity where no radio-marked birds occurred, but where we visually observed unmarked waterfowl on the ground. The mean kernel bandwidth distance of ground data that produced the strongest correlation between mean reflectivity and the kernel waterfowl density among winters was 5.0 km. Additionally, for all nights pooled across winters and radar sites (*n* = 55), the estimated mean median travel distance of birds from their departure location at a sun elevation of −5.0° based on radar observations of bird height distributions was 3.85±0.35 km, and the mean ± SE range (*a*) of spatial autocorrelation of radar reflectivity based on semivariogram analyses was 3.79±0.12 km.

## Discussion

Weather radar observations of the temporal patterns in crepuscular waterfowl activity were consistent with other published field observations and our own visual field observations. We attribute the initial post-sunset reduction in relative bird density aloft to the cessation of afternoon flights by geese, waterbirds, and songbirds returning to roosting sites. We observed primarily greater white-fronted goose and white-faced ibis returning after sunset to roosting sites within wetland habitats. Similarly, Ely [19] observed that greater white-fronted geese within the CVC typically end their afternoon feeding flights within 20 minutes after sunset. Blackbirds, *Agelaius* spp., also engage in afternoon feeding flights that end shortly after sunset within the CVC [42]. Based on the radar observations and verified by our visual field observations, nearly all the activity of roosting birds in the air subsided shortly before waterfowl initiated evening flights. Thus, the cessation of afternoon bird flights did not overlap with the onset of evening waterfowl feeding flight.

The mean initiation time of 23 min after sunset for waterfowl feeding flights based on radar observations fell within the range of initiation times reported by others [15], [16], [18], [20] (Table 1) and corresponded with our visual field observations of dabbling ducks initiating flight and leaving wetland habitats. Variability in the timing of evening flights among individual days relative to sun elevation has been associated with weather and sunlight conditions. For example, departures can be 10 to 15 min earlier under completely overcast conditions [18], or delayed a few minutes when bright moonlight is present [15], [20]. However, the standard deviation in the timing of evening flights that we observed among individual days was less than half the magnitude of the temporal sampling rate of the radar. Thus, sampling birds at a static sun elevation across nights would likely produce results similar to accounting for the variability in the timing of evening flights for individual days.

**Table 1:** Mean evening flight initiation time for wintering dabbling duck species at various locations.

Mean ± SE flight initiation time (min after sunset)	Species	Location	Source
22±1	northern pintail	Louisiana	[20]
∼25	northern pintail, American green-winged teal (*Anas carolinensis*)	Louisiana	[15]
25±2	Multiple: primarily northern pintail, mallard, American green-winged teal, American wigeon (*Anas americana*)	Texas	[18]
∼30	northern pintail	California	[16]

Although we observed non-waterfowl birds aloft during evening flights, their contribution to radar reflectivity measures is likely minimal. Waterfowl are much more abundant in the CVC during winter than other waterbirds (e.g., about 3–4 million ducks and 1 million geese [25] vs. about 300 000 shorebirds [43], and 30 000 sandhill cranes, *Grus canadensis* [44]) and most non-waterfowl birds are primarily diurnal feeders. However, we did observe black-crowned night-herons initiating flights concurrent with waterfowl evening flights. Seibert [45] observed that evening departure of night-herons from diurnal roosting sites coincides with the feeding flights of waterfowl. Non-waterfowl bird species including waterbirds aloft have been observed during similar winter evening waterfowl feeding flights in Louisiana but waterfowl comprised at least 97% of the birds within the radar beam [22].

We selected the sun elevation of −5° (28 min after sunset) as our target sampling time for quantifying the relative diurnal density of waterfowl at the ground. We based this primarily on the magnitude of the correlation between reflectivity and observed bird density on the ground and the high degree of spatial heterogeneity of the radar data. As expected, the strongest correlations occurred almost immediately after the initiation of evening flight and became weaker over time. Additionally, the associations of birds with their departure locations as they spread out into the air quickly dissipated as reflectivity became more spatially homogeneous (i.e., less “focused”) and autocorrelated to a greater distance over time. We doubt that an effective radar measure correction for the spreading nature of how waterfowl leave their departure locations is possible. In contrast, migrating landbirds leave their stopover sites in a relatively uniform direction, so adjustment for the displacement of birds aloft from their ground source is possible [10].

Maximum detection range of birds had little influence on our selection of a target sampling time. This is because the maximum detection range of birds was rather constant during the critical first 25 min after the initiation of flight, before increasing thereafter as evening flight progressed. During this time birds were likely engaged in a mixture of climbing, descending, and level flight due to individual variability in their flight timing and relatively-short travel distances to feeding sites, which resulted in relatively constant mean bird height in the air. Baldassarre and Bolen [18] observed that the evening departures of most individuals occurred within a 10 to 15 minute period. Additionally, evening flight distances for northern pintail and mallards within the northern CVC average 7.00±0.11 km and 3.60±0.06 km, respectively [25]. Combining these observations with the average flight speed of northern pintails during feeding flights (10.5 m/s) derived from Cox and Afton [20], we estimate the total mean duration of feeding flight activity of pintails and mallards to be 21 to 26 min and 16 to 21 min, respectively. This corresponds closely to the duration of the period of stable bird height distributions.

Some birds remained in the air for longer than the estimated duration of short-distance feeding flights by dabbling ducks based on our visual inspection of the time series of radar data. We propose the possibility that the radars observed two types of evening flight that were initiated concurrently; short-distance feeding flights and long-distance dispersal flights of birds. While screening radar data, we noticed the radars regularly detected distinct groups of birds moving long distances (e.g., 10's of kilometers) for up to two hours after the initiation of evening flights and well after the subsidence of short-distance feeding flights. This long distance/duration flight activity also corresponded to the increase in mean bird height in the air after the initial pulse of feeding flights subsided. Northern pintails [46], [47] and white-fronted geese [25] are known to move widely among basins throughout the CVC during winter. The long-distance flight observed by the radar may be those of waterfowl moving to other wintering areas within and outside of the CVC. A detailed analysis of this phenomenon was beyond the purview of our study but certainly warrants attention in future studies.

At our target sampling time, we found that radar reflectivity measured at the onset of nocturnal waterfowl flight was positively spatially related to the observed diurnal abundance of radio-marked waterfowl locations at the ground. Admittedly, the radio-telemetry dataset was not designed or collected for the purpose of ground-truthing radar observations. Consequently, the moderate correlations we found were likely constrained by the extent that our opportunistic dataset of radio-marked pintail and mallard distributions represented the distributions of all birds engaging in evening flights. We encourage the collection of more-robust ground-truthing data in the future through sampling of replicate areas of both high and low bird densities that are stratified throughout the radar coverage area. This would allow for assessing the accuracy and precision of weather radar observations for mapping waterfowl distributions with greater certainty. Regardless, these results compliment other studies that have found close temporal correlations between radar reflectivity and observed waterfowl density aloft within and across days [21], [22]. Thus, radar reflectivity can be used as a relative index of wintering waterfowl density for mapping waterfowl distributions across space and time.

An important limitation of weather surveillance radar for mapping bird distributions “on the ground” is the effective spatial resolution of the data, which is relatively coarse and not well suited for examining fine-scale or site-specific patterns [6]. It is important to consider the effective spatial resolution of radar data when deciding if weather radar data are appropriate for answering a particular research or resource management related question. The effective spatial resolution depends not only on the actual dimensions of sample volumes but also the accuracy and spatial heterogeneity of radar measures [8]. For example, our estimate of how far birds had traveled from their departure location when they were sampled aloft closely matched the scales at which kernel smoothing of ground waterfowl densities was most-closely correlated and to the radar data and at which radar data were spatially autocorrelated. Despite spatial autocorrelation out to about 4 km, finer-scale associations of birds with their departure locations can be resolved, particularly where discrete patches of suitable habitat are sampled by multiple radar sample volumes in an unsuitable matrix and when summarizing data across sampling nights. However, associations of birds from habitat patches or sites that are smaller than the physical dimensions of a single radar sample volume (mean = 19.0 ha, range = 3.5–34.5 ha) are likely not possible or potentially inaccurate due to the contribution of birds emanating from other areas within the sample volume to the total reflectivity value. In 2008, the resolution of low elevation angle radar measures for WSR-88D was improved 8-fold to 0.5°×250 m. Thus, the current “super-resolution” format allows for finer resolution of waterfowl distributions.

To the extent that our approach for using weather surveillance radars accurately and precisely maps waterfowl distributions has important advantages compared to traditional field-based approaches. Currently, annual estimates of regional wintering waterfowl abundance and distribution are obtained via aerial surveys. However, limited availability of trained observers and cost and danger of conducting aerial surveys may limit their future use. Data collected from human observers are subject to biases due to observer variability and bird visibility [48]–[50]. However, radar observations are not subject to these same biases. Radar measures are subject to their own suite of known biases primarily due to beam geometry and the distribution of birds in the air [8]. These measurement biases can be accounted for in large part using the approach developed in this study. Weather radars provide continuous and comprehensive coverage over large areas including remote areas that are difficult to access in the field. We found the radars could effectively detect waterfowl out to 83 km in regions with little topographic relief. This is similar to the detection range of 80 km when using WSR-88D to map landbird distributions during migratory stopover [10]. However, effective radar coverage for some areas may not extend to this range due to partial or complete blockage of the radar beam from topographic relief or human infrastructure near the radar site. Radar data are also freely accessible. Thus, radar data analysis is more cost effective than implementing large-scale field data collection through aerial or ground surveys, especially in cases where a relatively small field dataset can be used to calibrate or corroborate a more extensive radar dataset [6]. Finally, radar data have been archived back to the mid-1990's. Thus, retrospective analyses can be performed. Missing data in the archive may be of concern, particularly for radars operated by the Department of Defense (e.g., KBBX) from the earliest years. However, in recent years, we have experienced that the proportion of missing data from the archive for all radars has been significantly reduced to <3%.
